# Acute Hemolysis in the Emergency Department: Think about *Clostridium perfringens*!

**DOI:** 10.1155/2013/948071

**Published:** 2013-09-18

**Authors:** Roustit Cécilia, Vallé Baptiste, Clouzeau Benjamin, Heydel Virginie, Valdenaire Guillaume, Revel Philippe, Biais Matthieu

**Affiliations:** ^1^Department of Emergency Medicine, SAMU/SMUR, Pellegrin Medical University Hospital, 33 000 Bordeaux, France; ^2^Intensive Care Unit, Pellegrin Medical University Hospital, 33 000 Bordeaux, France

## Abstract

*Clostridium perfringens* (CP) gives several clinical settings, from an asymptomatic to a massive intravascular hemolysis. We report a case of fatal intravascular hemolysis due to CP septicemia having a hepatic supposed starting point in the emergency department. Like in many cases, the diagnosis was made when patient had already gone into shock and died. The CP septicemia often complicated the course of the digestive or genital pathologies. The alpha toxin can damage the structural integrity of the red cell membrane by means of a phospholipase activity. Nevertheless, a massive intravascular hemolysis arises only rarely in this septicemia, only from 7 to 15% of the cases. The emergency physician has to think about this complication in case of hemoglobinuria and/or signs of hemolysis associated with a septic syndrome. An immediate antibiotic treatment adapted as well as the symptomatic treatment of the spread intravascular coagulation could improve the survival of these patients.

## 1. Clinical Case

A 64-year-old man was brought to the emergency department by ambulance for faintness at home. He had history of diabetes, high blood pressure, and dyslipidemia. On arrival, the patient is alert but febrile (38.6°C/101.5 F). The initial vital signs showed tachycardia (109 per minute), blood pressure of 133/76 mmHg, a respiratory rate of 15 breaths/min, oxygen saturation 99% in room air and hyperglycemia of 2.70 g/L. The clinical examination was normal. The urinary tests find only a glycosuria without hemoglobin. The chest radiography and the ECG are without abnormalities.

The entrance biological results focus on an inflammatory syndrome: leukocytes count of 15.8 × 10^3^/*μ*L and C-reactive protein: 95 mg/L. Complete blood count revealed a hemoglobin level of 15.4 g/dL, a hematocrit of 46, and 3 T/L and a platelet count of 137 × 10^3^/*μ*L. The total bilirubin is up to 40 *μ*mol/L with hepatic cytolysis (serum aspartate transaminase ASAT: 114 U/L, serum alanine transaminase ALAT: 201 U/L, and a cholestasis *γ*-glutamyltransferase GGT: 253 U/L, alkaline phosphatase PAL: 146 U/L). The blood biochemistry analyses were normal.

The physician suggested bed rest, analgesics, and intravenous antibiotherapy with ceftriaxone. Ten hours later, the patient experienced acute dyspnea with polypnea up to 46 per min and oxygen desaturation of 80%. He was admitted to intensive care unit. He presented a hyperthermia up to 39.5°C, and blood pressure dropped to 80/60 mm Hg. He was resuscitated with 1 L of saline 9% through 2 peripheral lines. First blood gases showed metabolic acidosis. The focused chest sonography performed was normal. The urinary colorimetric bandelet finds a high hematuria level.

The department was called by the laboratory because the laboratory results were very hemolyzed: hemoglobin level: 9.2 g/dL, hematocrit (Ht) 11.1% (NR: 40 to 50%), blood platelets: 22 × 10^3^ *μ*/L, rhabdomyolysis creatinine phosphokinase (CK) 750 U/l, myoglobin: 1059 *μ*g/L, troponin: 0.14 ng/mL, acute renal insufficiency (urea: 14.8 mmol/L), creatinin: 165 *μ*mol/L, infectious syndrome (CRP: 102 mg/L), procalcitonin PCT: 37.38 *μ*g/L, and a severe hepatic failure (ALAT: 1022 U/L; ASAT: 1100 U/L, and GGT: 115 U/L).

Coagulation studies were in favour of a systemic intravascular coagulation (D-Dimers: 8805 ng/mL, TCA ratio: 1.99). The Coombs and fibrinogen tests were not possible because the hemolysis of sample, the ethanol test, and the haptoglobin dosing were not done.

Within minutes of this report, the patient collapsed. A pulseless electrical activity arrest quickly ensured. Nevertheless, the patient died shortly thereafter. No autopsy was undertaken because of the family's refusal. 48 h later, urinary cultures get back negative contrary to blood cultures that will come back positive for *Clostridium perfringens.*

## 2. Discussion

The *Clostridium perfringens* is a Gram+, anaerobic bacterium, from the commensal flora of the digestive and feminine genital tract. It has a strong proliferation proportion, by doubling in number within 7 minutes [1]. It secretes many toxins involved in the hemolysis. The alpha toxin can damage the structural integrity of the red cell membrane by means of a phospholipase activity [2]. This leads to spherocytosis and subsequent hemolysis.

The CP septicemias often complicates the course of the digestive or genital pathologies. Nevertheless, a massive intravascular hemolysis arises only from 7 to 15% of the cases [3].

In our case, considering medical history and positive cultures with CP, we conclude to CP septicemia with massive hemolysis with a biliary or hepatic supposed starting point. No focused abdominal sonography or abdominal X-ray was done in the ED although it had been requested.

For the ED physician, in case of intravascular coagulation, the additional tests to be made are reticulocytes (high), the test of Coombs, the blood smear, LDH (>1000), haptoglobin (collapsed), and free bilirubin (increased). 

The treatment of massive acute intravascular hemolysis due to CP has to include a premature antibiotic treatment (G or A penicillin), a correction of the disorders of the hemostasis, massive blood transfusion, or even an exsanguinotransfusion to check the hemolytic phenomena. Hemodialysis and hyperbaric oxygen therapy was already proposed as an adjuvant treatment.

A recent review of the literature was undertaken for 40 cases published since 1990, none having been published in emergency medicine literature [4]. Only diabetes seemed to be a risk factor of septicemia due to CP. Only 8 patients out of 40 survived with an 8-hour average time between the admission deadline and the death. As our patient's case, a quarter of similar case descriptions found no accurate infectious origin. 

 Massive intravascular hemolysis due to CP is a well-known complication which remains of little frequency. The emergency physician has to think about this complication in case of hemoglobinuria and/or signs of hemolysis associated with a septic syndrome. An immediate hospitalization in intensive care unit coupled with an antibiotic treatment adapted as well as the symptomatic treatment of the spread intravascular coagulation could improve the survival of these patients.
